# Primary renal diffuse large B-cell lymphoma presenting as new-onset kidney failure

**DOI:** 10.1007/s00467-025-06833-y

**Published:** 2025-06-10

**Authors:** Massimiliano Bertacchi, Lea Vasey, Alexandra Wilhelm-Bals, Anne-Laure Rougemont, Frederic Baleydier, Paloma Parvex

**Affiliations:** 1 Division of Pediatric Nephrology, Department of Pediatrics, Gynecology and Obstetrics, Geneva University Hospitals, Geneva, Switzerland; 2 Division of General Pediatrics, Department of Pediatrics, Gynecology and Obstetrics, Geneva University Hospitals, Geneva, Switzerland; 3 Division of Clinical Pathology, Diagnostic Department, Geneva University Hospitals, Geneva, Switzerland; 4 Division of Pediatric Oncology and Hematology, Department of Paediatrics, Gynaecology and Obstetrics, Geneva University Hospitals, Geneva, Switzerland; 5 CANSEARCH Research Platform for Pediatric Oncology and Hematology, Department of Pediatrics, Gynecology, and Obstetrics, Faculty of Medicine, University of Geneva, Geneva, Switzerland

**Keywords:** Primary renal lymphoma, Dialysis, Pharmacology, Personalized medicine

## Abstract

**Background:**

Primary renal lymphoma is a rare form of kidney tumor, accounting for less than 1% of all kidney masses, and with only a few pediatric cases reported in the literature. Bilateral forms can present with kidney failure, constituting a therapeutic challenge for both hematologists and nephrologists, with the need to combine complex chemotherapy regimens and kidney replacement therapy.

**Methods:**

We present a rare case of primary bilateral renal diffuse large B-cell lymphoma in a teenage girl presenting with kidney failure (eGFR of 7 ml/min/1.73 m^2^), proposing a practical approach to optimize chemotherapy on kidney replacement therapy. We discuss diagnostic methods, the management and adjustment of chemotherapy and hyperhydration protocols, and the tailoring of highly efficient hemodiafiltration sessions to grant sufficient drug exposure while avoiding drug accumulation and toxicity in the context of kidney failure.

**Results:**

This approach permitted a substantial reduction of chemotherapy adverse effects while inducing remission and partial recovery of kidney function in our patient, with hemodialysis discontinuation after 6 months, and an eGFR that had improved to 28 ml/min/1.73 m^2^ at 12 months.

**Conclusions:**

Managing complex chemotherapy protocols in kidney failure can be challenging, and the collaboration of a multidisciplinary team is essential. The adjustment of drug dosing and the tailoring of hemodialysis can increase patients’ tolerance while maintaining chemotherapy efficiency.

**Graphical abstract:**

**Figure:**
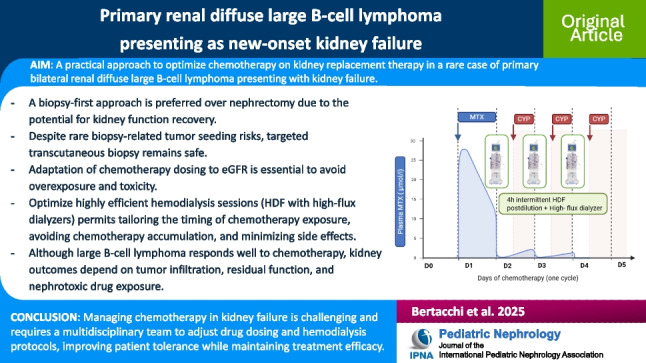
A higher resolution version of the Graphical abstract is available as Supplementary information

**Supplementary Information:**

The online version contains supplementary material available at 10.1007/s00467-025-06833-y.

## Introduction

In adults, kidney involvement of non-Hodgkin lymphoma (NHL) occurs frequently, with diffuse large B-cell lymphoma (DLBCL) originating from a nodal or extra nodal mass accounting for about 30% of those cases. Primary renal lymphoma (PRL) is a rare condition due to the absence of lymphoid tissue in the kidney and is mostly reported in adults over 60 years of age [1]. However, DLBCL represents only 1% of all kidney masses in adults, and only a few cases of PRL have been reported in children [2, 3]. Although there are few differential diagnoses of bilateral kidney tissular masses in children and adolescents, the histological confirmation of renal lymphoma is crucial to exclude renal cell carcinoma (RCC), a rare differential diagnosis of isolated kidney mass primarily treated with surgery instead of chemotherapy. As PRL is usually unilateral, kidney failure is exceptional, but acute or chronic kidney injuries have been reported, and an improvement in kidney function was observed after chemotherapy in adults and children [3–5]. Bilateral forms can result in severe kidney failure, requiring kidney replacement therapy, and represent a great challenge for the management of chemotherapy. This article proposes a practical approach to optimize chemotherapy in kidney failure, tailoring hemodialysis to optimize drug exposure and reduce toxicity.

## Case diagnosis

We present the case of a 13-year-old girl presenting with asthenia and weight loss over four months. Clinical examination revealed arterial hypertension (> P95) and a palpable abdominal mass. She had no other complaints and no fever, pain, or edema. She was previously healthy without a history of hospitalization or receiving any medication. The family history was negative for chronic diseases.

An initial workup revealed non-regenerative anemia with a Hb 107 g/l and severe kidney failure with a creatinine of 756 µmol/l (8.55 mg/dl) and cystatin-C of 4.29 mg/l, corresponding to an eGFR of 7 ml/min/1.73 m^2^ according to CKiD-U25-creatinine. Urea was 42 mmol/l, K 6.3 mmol/l, and phosphate 2.06 mmol/l. She had severe metabolic acidosis (pH 7.28 Kpa, pCO2 31.5 mmHg, HOC3^−^ 14 mmol/l). Serum LDH was elevated (453 U/l, Normal < 265 U/l), as serum albumin (45 g/l, Normal 35–48 g/l). Urine analysis showed nephrotic range proteinuria with a protein/creatinine ratio of 427 mg/mmol (3.8 mg/mg), without microhematuria. An extended infectious workup was performed, including PCR for CMV, EBV, and BKV, as well as HBV, HCV, syphilis, and HIV serologies, all with negative results.

**Fig. 1:**
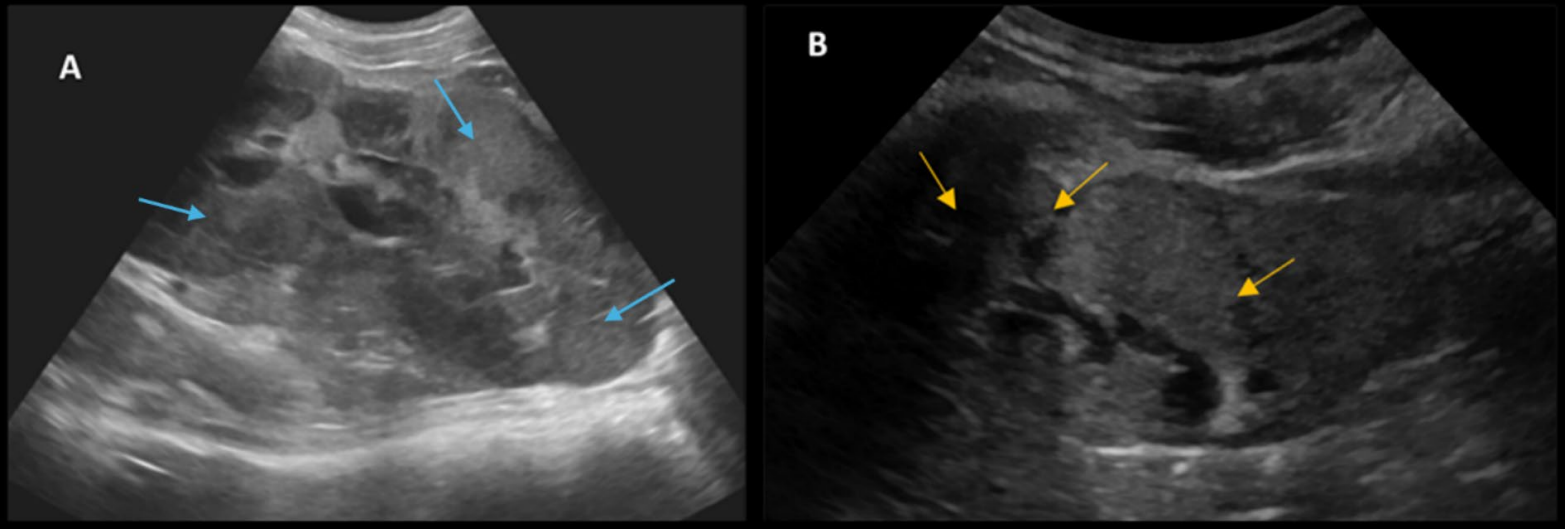
Kidney ultrasound **A** at diagnosis showed bilateral nephromegaly (left kidney 19 cm; right kidney 18 cm) with reduced corticomedullary differentiation and a diffuse nodular infiltrate (blue arrows) and **B** at 12 months after the end of therapy, with reduced corticomedullary differentiation and reduced size (left kidney 9 cm and right kidney 6.7 cm) and the presence of multiple hypoechogenic scar tracts (yellow arrows)

**Fig. 2:**
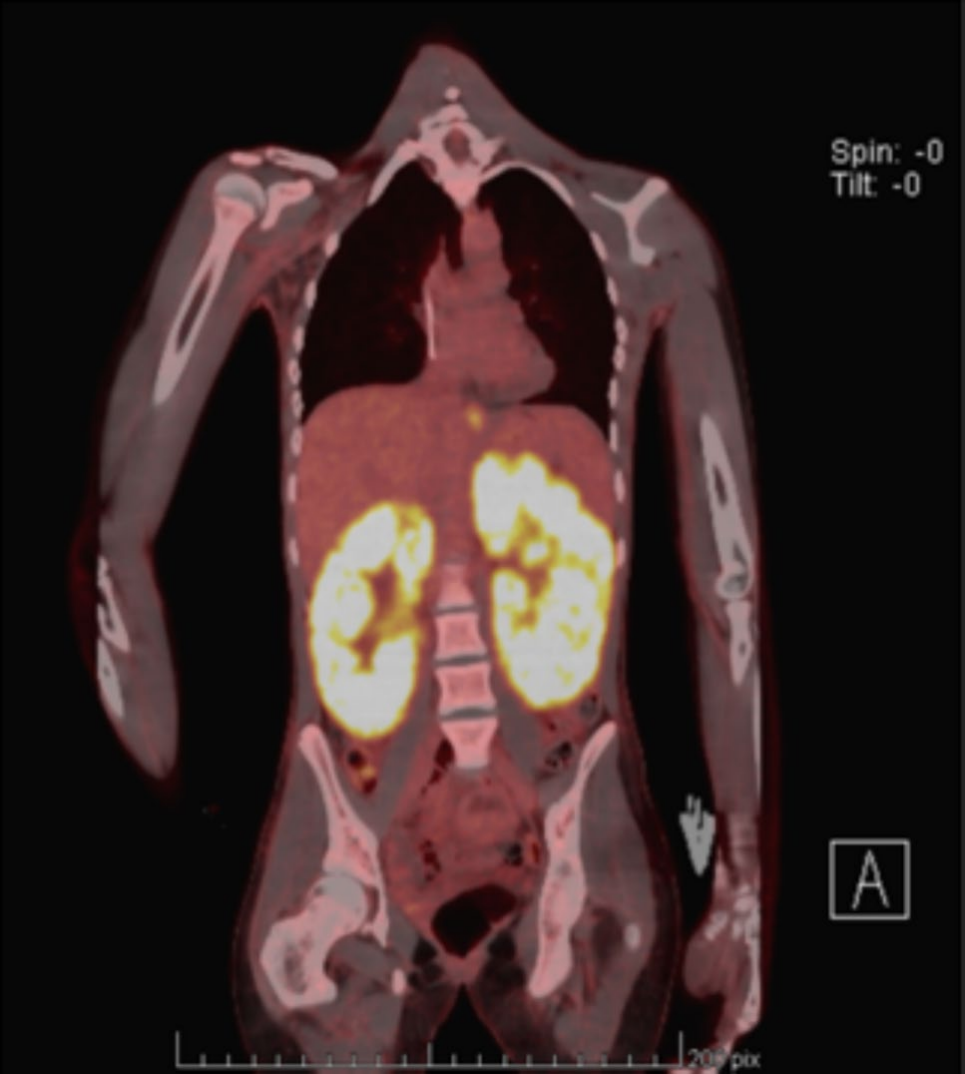
FDG-PET/CT scan showed bilateral enlarged hypermetabolic kidneys without extrarenal hypermetabolic region

**Fig. 3:**
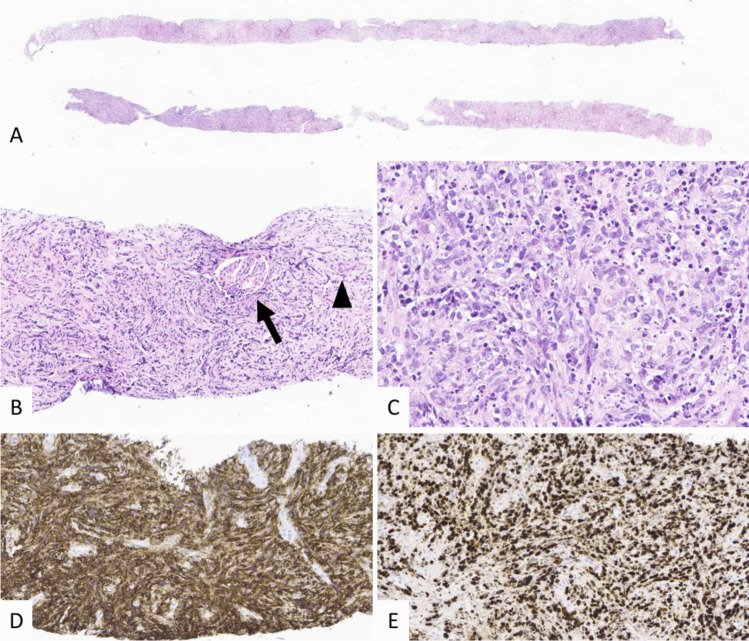
**A**–**C** Biopsy of the kidney demonstrates infiltration by numerous large cells encasing a glomerulus (arrow) and a tubule (arrowhead). The tumor cells exhibit large nuclei with occasional prominent nucleoli, and frequent apoptosis is observed (Hematoxylin Eosin, H&E). **D** Tumor cells express the B-cell marker CD20. **E** The proliferation index, assessed by MIB-1 (Ki-67), is markedly elevated at approximately 95%

Kidney ultrasound showed two large kidneys with a bilateral diffuse nodular infiltrate (Fig. 1A). A PET scan was performed and revealed bilateral nephromegaly (left kidney 19 cm; right kidney 18 cm; both > P95) with high metabolic activity without any extrarenal hypermetabolic region (Fig. 2). A percutaneous targeted nodular biopsy confirmed high-grade CD20+ diffuse large B-cell lymphoma (DLBCL) with no rearrangement on cytogenetics (Fig. 3). After staging evaluation, in the absence of bone marrow or CNS spreading, the isolated kidney hypermetabolism on the FDG-PET/CT scan confirmed the diagnosis of PRL.

The patient started standard kidney failure treatment with chronic hemodialysis, bicarbonate substitution, potassium-binding resins, calcitriol, and erythropoietin.

## Chemotherapy and dialysis challenge

Our patient was treated according to the international inter-B-NHL Ritux 2010 (group B) protocol [6, 7] with rituximab combined with sequential courses of steroids and chemotherapy. The therapeutical challenge was to combine sufficient drug exposure while avoiding drug accumulation and toxicity secondary to reduced kidney clearance. Chemotherapy was then adapted for an eGFR < 10 ml/min/1.73 m^2^, based on the recommendations of Ashley and Dunleavy (The Renal Handbook, CRC Press 2019). The proposed adaptation of chemotherapy drugs and the impact of hemodialysis on drug clearance are summarized in Table 1.

**Table 1:** Summary of the Inter-B-NHL Ritux 2010 (group B) protocol: adaptation of chemotherapy drugs and impact of hemodialysis on drug clearance

Course of treatment	Standard daily doses according to Inter-B NHL Ritux 2010	Adjusted doses delivered according to recommendations for eGFR < 10 ml/min/1.73 m^2^	Dialysis impact
Off-protocol priming steroid pre-phase (before R-COP) *			
- Prednisone	60 mg/m^2^ day 1–6	- No adaptation	- Not dialyzed
R-COP *			
- Rituximab	375 mg/m^2^ day 6 (= day-2 1^st^COPADM)	- Full dose	- Not dialyzed
- Cyclophosphamide	300 mg/m^2^ day 1	- Full dose keeping to the 12 h recommended before the next dialysis session	- Dialyzed, exposure of at least 12 h
- Vincristine	1.0 mg/m^2^ day 1	- Full dose	- Not dialyzed
- Prednisone	60 mg/m^2^ day 1–7	- Full dose	- Not dialyzed
- Intrathecal MTX/methylprednisolone	15 mg/3 mg day 1	- Full dose	- Unknown
R-COPADM 1 and 2 *			
- Rituximab	375 mg/m^2^ day-2 and day 1	- Full dose	- Not dialyzed
- Cyclophosphamide	250 mg/m^2^ 12 hourly day 2–4	- 50% dose reduction keeping to the 12 h recommended before the next dialysis session	- Dialyzed, exposure of at least 12 h
- Vincristine	2.0 mg/m^2^ (caped to 2.0 mg) day 1	- Full dose	- Not dialyzed
- Prednisone	60 mg/m^2^ day 1–5	- Full dose	- Not dialyzed
- Doxorubicin	60 mg/m^2^ day 2	- 75% dose reduction	- Not dialyzed
- Methotrexate^§^	3000 mg/m^2^ day 1	- 50% dose reduction keeping to the 12 h recommended before the next dialysis session	- Dialyzed, exposure of at least 24 h
- Intrathecal MTX/methylprednisolone	15 mg/3 mg day 1 and day 6	- 50% reduction dose for MTX (arbitrary)	- Unknown
R-CYM 1 and 2 *			
- Rituximab	375 mg/m^2^ day-2 and day 1	- Full dose	- Not dialyzed
- Cytarabine	100 mg/m^2^ 24 h continuous infusion day 2–6	- Full dose	- Not dialyzed
- Methotrexate^§^	3000 mg/m^2^ day 1	- 50% dose reduction keeping to the 12 h recommended before the next dialysis session	- Dialyzed, exposure of at least 24 h
- Intrathecal MTX/methylprednisolone	15 mg/3 mg day 2	- 50% reduction dose for MTX (arbitrary)	- Unknown
- Intrathecal cytarabine/methylprednisolone	30 mg/3 mg day 7	- Full dose	- Unknown

^*^Body surface was calculated based on the initial dry weight

^§^The rescue with folinic acid was applied as per protocol as follows: folinic acid 15 mg/m^2^ 6 hourly from H24 to plasma concentration of methotrexate (assessed every 24 h) less than 0.15 micromol/L

**Fig. 4:**
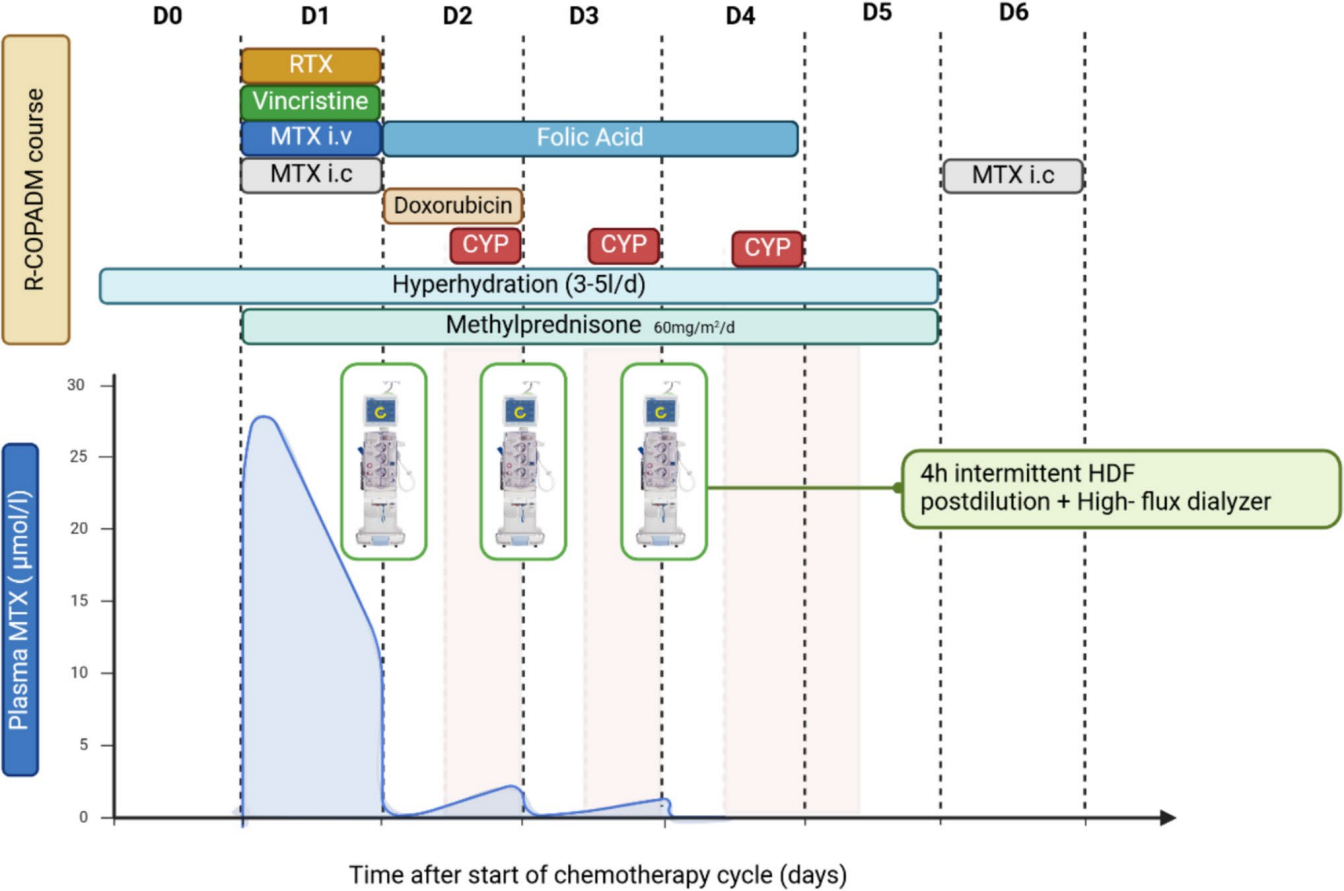
R-COPADM course with the implementation of highly efficient HDF q24 after MTX IV was administered. HDF was repeated until complete clearance of MTX rebound levels (3 sessions). CYP was administered 12 h before the next HDF session to grant the minimal exposure time needed. In the corner, the FDG-PET scan shows the two hypermetabolic enlarged kidneys without extrarenal foci. The curve of MTX plasma concentration was estimated based on MTX peak levels and residual post-HDF levels. The image was generated with a licensed version of BioRender.com

Highly efficient hemodiafiltration (HDF) with high-flux dialyzers was tailored to grant an exposure of at least 24 h for MTX and 12 h for CYP. HDF was then repeated following residual MTX blood level until complete clearance (target < 0.15). We chose high blood flow (7 ml/kg/min) and high dialysate flow (10–15 ml/kg/min) over 4-h sessions to maximize MTX clearance (Fig. 4). Standard hyperhydration (2000 ml/m^2^) in the context of chemotherapy was adapted according to the patient’s residual diuresis (ca. 2000 ml/d) and the tolerated HD ultrafiltration (between 1000–1800 ml over a 4-h session) to avoid fluid overload.

## Discussion

Even if the differential diagnosis of a primary bilateral kidney mass is narrow, comprehensive laboratory investigations and radiological imaging, including ultrasound and cross-sectional imaging, are needed to clarify the diagnosis and cause of kidney failure and to choose the right therapy. Early involvement of hemato-oncologists is essential to discuss investigations and adapt chemotherapy protocols. If feasible, we recommend considering the use of extended kidney function biomarkers such as cystatin-C and inulin or iohexol clearance to better assess kidney function in oncologic settings with possible secondary sarcopenia. An early full-body PET scan is essential to determine extrarenal involvement. Finally, as opposed to a significant number of adult patients diagnosed after unilateral radical nephrectomy, a biopsy-first approach is preferred in children because of the excellent prognosis of PRL after chemotherapy and the potential improvement of kidney function.

Even though isolated cases of tumor seeding in the tract of kidney tumor biopsy have been described [8, 9], performing a transcutaneous targeted biopsy is safe in this setting, with a minimal combined complication risk of 1.6% (tumor bleeding, rupture, and needle track recurrence) [10]. Following the guidance offered by the UMBRELLA protocol 2016 for childhood kidney tumors, indications of targeted tumoral biopsy are focused on unusual clinical, biological, or radiological findings (listed in Supplementary Table 1). There are only a few formal contraindications, mostly regarding young infants (6 months old or younger) or completely cystic tumors, in which an upfront surgery is preferable. The UMBRELLA protocol recommends performing transcutaneous targeted biopsy under general anesthesia with radiological guidance (ultrasound or CT). To avoid complications and tumor seeding, co-axial technique and retroperitoneal access are recommended [8, 10]. Procedural recommendations according to the UMBRELLA protocol are reported in Supplementary Table 1.

An accurate assessment of chemotherapy drugs’ pharmacokinetic and pharmacodynamic properties and their dialyzability permits therapy optimization. Tailoring hyperhydration and dialysis parameters permits the treatment of kidney failure while modulating chemotherapy exposure, reducing side effects. In our case, an initial suboptimal systemic MTX clearance due to delayed HDF initiation resulted in sustained medullary suppression and hemorrhagic mucositis. The protocol was optimized with consecutive HDF q24 until complete clearance of MTX (following rebound levels), strongly reducing adverse effects of the following cycles while maintaining optimal drug exposure (Fig. 4).

Although large B-cell lymphoma typically responds well to chemotherapy, there are limited data on the long-term kidney outcomes for primary kidney forms presenting with kidney failure. Several factors can affect kidney outcomes, including residual kidney function at diagnosis, the extent of tumoral infiltration, and cumulative exposure to nephrotoxic chemotherapy [3]. We recommend close monitoring of LDH plasma levels, and repeated kidney ultrasound is required every 3 months for the first 2 years post-treatment and then every 6 months for the next 3 years. If a relapse is suspected based on ultrasound findings, a PET scan is recommended, and if abnormalities are detected on the PET scan, a targeted biopsy should be repeated.

Our patient remained in complete remission with a negative PET scan 1 year after the end of treatment. Slow recovery of kidney function permitted pharmacological management of electrolyte and acid–base disorders so that hemodialysis could be stopped after 6 months. Kidney function slowly recovered to an eGFR of 28 ml/min/1.73 m^2^ at 12 months. She started regular sports activities without complaints. Medication was discontinued except for low doses of calcium channel blockers for hypertension. An ultrasound at 12 months revealed kidney asymmetry with multiple scars (Fig. 1B), which was confirmed by a DMSA scintigraphy and indicated permanent damage to both kidneys. This clinical evolution was comparable to the case of a 4-year-old reported by South [3] showing partial recovery of kidney function to an eGFR of 50 ml/min/1.73 m^2^ at 6 months.

In conclusion, even if the management of oncologic patients with concomitant kidney failure needing kidney replacement therapy is challenging, nephrologists should be aware of the potential synergistic effects of adapting chemotherapy protocols while performing tailored kidney replacement therapy and managing chemotherapy exposure while treating kidney failure.

Our case shows that an adapted combination of immuno-chemotherapy is safe and efficient in treating primary renal lymphoma under kidney replacement therapy and that optimizing hemodialysis protocols can improve therapy tolerance.

## Summary

### What is new?

- We present a rare case of a 13-year-old girl with bilateral primary renal lymphoma resulting in kidney failure. We propose a rational approach for managing concomitant chemotherapy and hemodialysis.

## Supplementary Information

Below is the link to the electronic supplementary material.

Graphical abstract (PPTX 161 KB)

Supplementary file1 (DOCX 20 KB)

## Data Availability

All data generated or analyzed during this study are included in this article and its supplementary information files.
