# Supplementary material for: Primary renal diffuse large B-cell lymphoma presenting as new-onset kidney failure
Source: Pediatr Nephrol. 2025 Jun 10;40(10):3181–6. doi: 10.1007/s00467-025-06833-y (PMC12401750; doi:10.1007/s00467-025-06833-y)
Supplement: Supplementary file 1 — Graphical abstract (PPTX 161 KB) [file 467_2025_6833_MOESM1_ESM.pptx]

## Slide 1
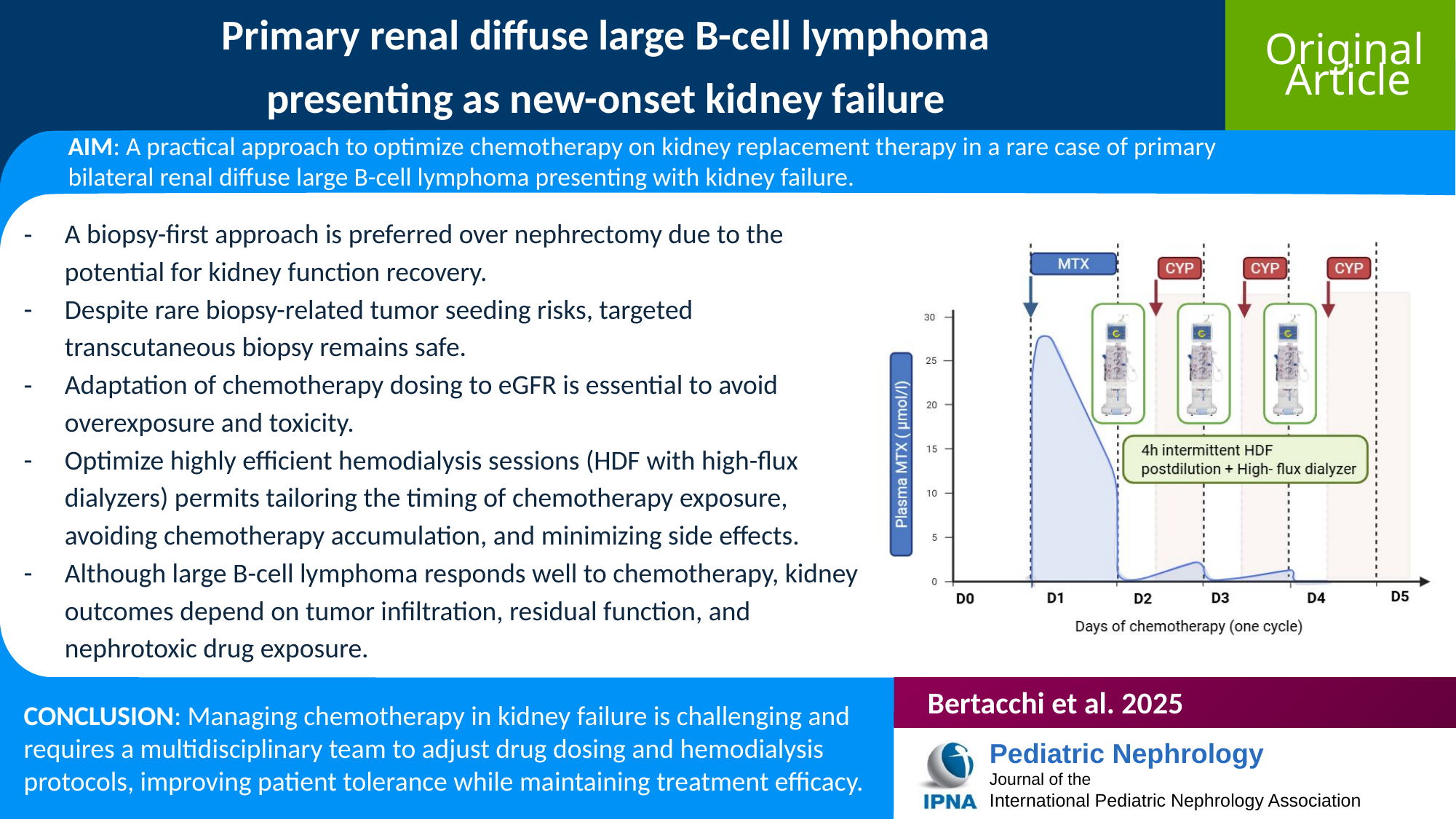

Primary renal diffuse large B-cell lymphoma
presenting as new-onset kidney failure
AIM: A practical approach to optimize chemotherapy on kidney replacement therapy in a rare case of primary bilateral renal diffuse large B-cell lymphoma presenting with kidney failure.
A biopsy-first approach is preferred over nephrectomy due to the potential for kidney function recovery.
Despite rare biopsy-related tumor seeding risks, targeted transcutaneous biopsy remains safe.
Adaptation of chemotherapy dosing to eGFR is essential to avoid overexposure and toxicity.
Optimize highly efficient hemodialysis sessions (HDF with high-flux dialyzers) permits tailoring the timing of chemotherapy exposure, avoiding chemotherapy accumulation, and minimizing side effects.
Although large B-cell lymphoma responds well to chemotherapy, kidney outcomes depend on tumor infiltration, residual function, and nephrotoxic drug exposure.
Bertacchi et al. 2025
CONCLUSION: Managing chemotherapy in kidney failure is challenging and requires a multidisciplinary team to adjust drug dosing and hemodialysis protocols, improving patient tolerance while maintaining treatment efficacy.
