# Supplementary material for: Primary renal diffuse large B-cell lymphoma presenting as new-onset kidney failure
Source: Pediatr Nephrol. 2025 Jun 10;40(10):3181–6. doi: 10.1007/s00467-025-06833-y (PMC12401750; doi:10.1007/s00467-025-06833-y)
Supplement: Supplementary file 2 — Supplementary file1 (DOCX 20 KB) [file 467_2025_6833_MOESM2_ESM.docx]

**Primary renal diffuse large B-cell lymphoma presenting as new-onset kidney failure**

**Supplementary Table 1.** UMBRELLA Protocol - Indications, limitations, and contraindications of Cutting Needle Biopsy

| **Indications:**  Cutting needle biopsies should be considered by: | **Unusual clinical presentations:**  • Age > 6 years  • Urinary infection or septicemia  • Psoas infiltration  • Pulmonary metastasis < age of 2 years (suspicious for MRTK)  • Extra-hepatic and extra-pulmonary metastases |
| --- | --- |
|  | **Unusual findings by imaging:**  • Numerous calcifications  • Voluminous lymphadenopathies  • Renal parenchyma not visible  • Almost totally extra-renal process |
|  | **Biological findings**  • Hypercalcemia (suspicious for MRTK)  • LDH level > 4N (suspicious for neuroblastoma or malignant hemopathy) |
| **Limitations**:  Cutting needle biopsies are of limited use in the differentiation of: | • Nephroblastoma vs. Nephroblastomatosis  • Diffuse anaplasia vs. focal anaplasia  • Stromal subtype vs. embryonal rhabdomyosarcoma  • Cystic Nephroma, cystic partially differentiated nephroblastoma (CPDN) vs. cystic nephroblastoma |
| **Contraindication:**  Cutting needle biopsies should not be used in: | • Age 6 months and younger (upfront surgery)  • Completely cystic tumors (consider upfront surgery) |
| **Procedural Recommendations** | • Under general anesthesia  • Ultrasound or CT guidance – make sure to sample solid and viable tumors, avoid sampling of necrotic or cystic areas  • Co-axial technique is mandatory  • Retroperitoneal biopsy tract only (do not use transperitoneal access)  • Use cutting needles with a size of 18 or 16 Gauge to guarantee sufficient tissue for pathologic differentiation.  • No direct fixation of all specimens (part of specimens in culture medium like RPMI or immediate freezing) |

Indications, limitations and contraindications of Cutting Needle Biopsy

Adapted from: UMBRELLA Protocol SIOP 2016 243
